# A Comparison of Production Performance, Egg Quality, and Cecal Microbiota in Laying Hens Receiving Graded Levels of Vitamin B_12_

**DOI:** 10.3389/fvets.2021.712183

**Published:** 2021-10-21

**Authors:** Rui Wang, Yan Bai, Yu Yang, Xiaotian Wu, Ruirui Li

**Affiliations:** ^1^Laboratory of Poultry Production, College of Animal Science, Shanxi Agricultural University, Taigu, China; ^2^Department of Life Sciences, Luliang University, Luliang, China

**Keywords:** vitamin B_12_, laying hens, production performance, eggshell quality, cecal microbiota

## Abstract

The objective of the study was to investigate the effect of fortified diets with standard vs. high levels of vitamin B_12_ on cecal microbiota composition, production performance, and eggshell quality of laying hens. Dietary treatments consisted of a basal diet with no supplementation of vitamin B_12_ or supplemented with 25, 100, and 400 μg/kg vitamin B_12_, respectively. A total of 432 laying hens were randomly assigned to four treatments with six replicates per treatment. No significant effect of dietary treatments on the production performance of hens was detected. The shell thickness of eggs from hens fed diet supplemented with 100 μg/kg of vitamin B_12_ was higher (*P* < 0.01) than that of eggs from hens fed control diet or supplemented with 25 μg/kg vitamin B_12_. The shell percentage of eggs from hens fed diet supplemented with 400 μg/kg of vitamin B_12_ was higher (*P* < 0.01) than that of eggs from hens fed other treatment diets. Dietary vitamin B_12_ did not modulate diversity of the cecal microbiota of the layers. At genus level, the cecal content from layers fed diet with supplemental level of 100 or 400 μg/kg of vitamin B_12_ had higher (*P* < 0.01) abundance of *Faecalibacterium* and lower (*P* < 0.05) abundance of *Acinetobacter* compared with the cecal content from layers fed other two diets. The abundance of *Lactobacillus* in the cecal samples from layers fed 100 μg/kg of supplemental level of vitamin B_12_ was higher (*P* < 0.05) than that from layers fed other three diets. The abundance of *Butyricicoccus* was higher (*P* < 0.05), while *Bilophila* was lower (*P* < 0.05) in the cecal content of layers fed 400 μg/kg of vitamin B_12_ diet compared with those from layers fed other three diets. The results of PICRUSt analysis indicated that 10 predicted metabolic functions of the cecal microbial communities were positively correlated to dietary vitamin B_12_ level. Overall, dietary supplementation of 100 or 400 μg/kg of vitamin B_12_ had equivalent effects and caused the significant change in composition and metabolic functions of cecal microorganisms, which could positively impact eggshell quality, metabolism, and gut health of laying hens.

## Introduction

Vitamin B_12_ is one of essential B vitamins required by humans and animals. Since it is involved in nucleic acid synthesis, carbohydrate and fat metabolism, and methyl synthesis, the deficiency of vitamin B_12_ will cause anemia, muscle weakness, severe neurologic problems, and many other symptoms in humans and animals (1–6). In laying hens, numerous studies indicated that vitamin B_12_ is required for optimal egg production, egg weight, and hen weight (7–11). Today, vitamins are commonly over fed in poultry feed to give safety margin for the deterioration of many vitamins during feed processing and storage (12–14). Also, vitamin-enriched eggs produced through supplementing extremely a high level of vitamins of interest to the layer diet for human consumption have been widely accepted (15–20). Subsequently, a significant portion of vitamins that was not absorbed in the small intestine of layers could reach the hind gut and affect the microbiota composition of the cecum. Giannella et al. reported that excess vitamin B_12_ in the intestine changed the gut microbiome composition and caused the overgrowth of intestinal bacterial (21). Degnan et al. found that the competition and exchange of vitamin B_12_ among microbes can regulate the gut microcommunity (22). It was documented that vitamin B_12_ and some other B vitamins changed the metabolism of microbiota, promoted bacterial colonization in the gut, and modulated bacterial virulence and the host defense to the pathogen infection (23–25). Results from *in vitro* and *in vivo* trials indicated that vitamin B_12_ was essential for some enteropathogens to utilize ethanolamine, which enhanced *Salmonella typhimurium* growth and its virulence gene expression (26–29). Xu et al. indicated that vitamin B_12_ supplementation changed microbial composition and increased the amount of short-chain fatty acids in an *in vitro* colon simulation (30).

Thus far, not much information is available for the link between high dietary vitamin B_12_ and the intestinal microbiota composition in poultry. Therefore, the purpose of the current study was to investigate the effect of fortified diets with standard vs. high levels of vitamin B_12_ on cecal microbiota composition and production performance of laying hens.

## Methods and Materials

All experimental procedures were carried out in accordance with the Guidelines of the Shanxi Agricultural University Animal Experiment Ethics Committee, and the license number was SXAU-EAW-2017-002Chi.001. The experiment was performed in the Animal Production Laboratory of Shanxi Agricultural University and Xingmin Animal Husbandry Industry Cooperative located in Taigu District of Jinzhong City.

### Animals, Diets, and Experimental Design

Based on a single-factor experimental design, a total of 432 healthy Jinghong No. 1 laying hens with the same initial body weight (BW) at 30 weeks of age were randomly assigned to four dietary treatments to give six replicate cages of three hens per cage. Dietary treatments included (1) corn soybean meal basal (basal) diet with no supplementation of vitamin B_12_ (B0), (2) basal diet supplemented with 25 μg/kg of vitamin B_12_ (B25), (3) basal diet supplemented with 100 μg/kg of vitamin B_12_ (B100), and (4) basal diet supplemented with 400 μg/kg vitamin B_12_ (B400). The commercial product used in this trial contained 1% vitamin B_12_ and was provided by Hebei Yuxing Bio-Engineering Co. Ltd. No vitamin B_12_ was detected in the basal diet by using high-performance liquid chromatography. This is because vitamin B_12_ is well-known to be the sole vitamin that is absent from plant-based feed sources. Three dietary supplemental levels of vitamin B_12_ included one standard level of 25 μg/kg recommended by breeders and vitamin-producing companies (31, 32), and two (100 or 400 μg/kg) high levels were used for producing vitamin B_12_-enriched eggs (15, 16). Other nutrient level of basal diet was designed based on the NRC recommendation (33). The composition and calculated nutrient level of basal diet are listed in Table 1.

**Table 1:** Ingredients and composition of the basal diet (as fed basis).

**Ingredient**	**%**	**Nutrient^c^**	**Value**
Corn	64.50	Metabolizable energy (MJ/kg)	11.08
Soybean meal	21.00	Crude protein (%)	16.46
Cottonseed meal	1.00	Crude fiber (%)	3.00
Linseed meal	2.00	Ether extract (%)	2.89
Limestone	9.66	Ash (%)	12.61
NaCl	0.30	Calcium (%)	3.51
*DL*-methionine (98%)	0.40	Total phosphorus (%)	0.50
L-Lysine·H_2_SO_4_ (70%)	0.04	Available phosphorus (%)	0.22
Choline chloride (50%)	0.11	Methionine (%)	0.39
Vitamin premix^a^	0.04	Lysine (%)	0.78
Mineral premix^b^	0.20	Threonine (%)	0.58
Calcium hydrophosphate	1.00	Methionine + Cysteine (%)	0.66
Phytase (5,000 IU/g)	0.02		
Total	100.00		

a *Supplied per kg diet: VA 14,400 IU, VD_3_ 54,00 IU, VK_3_ 3.2 mg, VE 32 mg, VB_1_ 2.4 mg, VB_2_ 10 mg, VB_6_ 4 mg, folate 1 mg, nicotinic acid 48 mg, pantothenic acid 14 mg, and biotin 0.16 mg.*

b *Supplied per kg diet: Cu (as copper sulfate) 8 mg, Fe (as ferrous sulfate) 50 mg, Mn (as manganese sulfate) 100 mg, Zn (as zinc sulfate) 90 mg, I (as potassium iodide) 0.40 mg, Se (as sodium selenite) 0.36 mg, and Co (as cobalt sulfate) 0.26 mg.*

c *The values of metabolizable energy, available phosphorus, and amino acids are calculated, and others are measured values*.

The experimental chicken coop was completely enclosed with the stainless-steel galvanized cages of $47_{*}$ $37_{*}$ 38 cm in size. In a 14-day pretrial period, all laying hens were fed a basal diet. Then the treatment diets were provided to the laying hens for another 42 days. The layers were free to eat feed and drink water during the entire pretrial and trial periods. The daily light exposure was 16 h with an intensity not <15 lx/m^2^.

### The Assay of Production Performance, Egg Quality, and Serum Indicators of Laying Hens

Hens in each replicate were weighed in-group at the start and at the end of the experiment to evaluate the BW change. Egg number, egg weight, and dead birds were recorded daily, whereas feed consumption was recorded weekly to calculate hen day egg production (%), feed intake (g/hen/day), egg mass (g/day), and feed conversion ratio (feed/egg mass).

A total of 18 egg samples (three eggs per replicate) were taken weekly in a 6-week trial period to do the analysis of egg weight and eggshell quality. Egg diameter was measured with a digital display vernier caliper (S102-101-101, SMCT, Shanghai). Eggshell strength was evaluated using an Egg Force Reader (EFR-01, Orka Food Technology Ltd., Israel). Eggshell thickness was measured on the large end, equatorial region, and small end, respectively, using an eggshell thickness gauge (Robotmation Co., Ltd., Tokyo, Japan), and the average value was calculated as the eggshell thickness measurement. The egg weight, egg yolk color, and Haugh unit (HU) were evaluated using an Egg Analyzer (EA-01, Orka Food Technology Ltd., Israel). Egg white and yolk were separated carefully, then the egg yolk and eggshell were weighed, and the ratio of the egg yolk, egg white, and eggshell was calculated.

Blood samples were taken from six hens per treatment (one bird per cage) to test the biochemical indicators of serum. Vitamin B_12_, progesterone, and estrogen levels were measured using enzyme-linked immunoassay kit (Shanghai Hushen Biological Technology Co., Ltd., China). Total cholesterol (TC) and triglyceride (TG) levels were measured using GPO-PAP (NanJing JianCheng Bioengineering Institute, China).

### The Assay of Cecal Microbiome

At the end of the experiment, one bird with a BW close to the mean BW of each cage was euthanized by cervical dislocation to collect the cecal content. A total of six samples per treatment were collected. After collection, the samples were immediately frozen by liquid nitrogen and preserved at −80°C until analysis. The frozen samples were used for isolation of metagenomic DNA.

#### DNA Extraction

Total bacterial genomic DNA samples were extracted using the Fast DNA SPIN extraction kits (MP Biomedicals, Santa Ana, CA, USA), following the instructions of the manufacturer, and stored at −20°C prior to further analysis. The quantity and quality of extracted DNAs were measured using a NanoDrop ND-1000 spectrophotometer (Thermo Fisher scientific, Waltham, MA, USA) and agarose gel electrophoresis, respectively. 16S rDNA amplicon pyrosequencing PCR amplification of the bacterial 16S rRNA gene V3–V4 region was performed using the forward primer 338F (5′-ACTCCTACGGGAGGCAGCA-3′) and the reverse primer 806R (5′-GGACTACHVGGGTWTCTAAT-3′). Sample-specific 7-bp barcodes were incorporated into the primers for multiplex sequencing. The PCR components contained 5 μl of Q5 reaction buffer (5×), 5 μl of Q5 High-Fidelity GC buffer (5×), 0.25 μl of Q5 High-Fidelity DNA Polymerase (5 U/μl), 2 μl (2.5 mM) of dNTPs, 1 μl (10 μM) of each forward and reverse primer, 2 μl of DNA template, and 8.75 μl of ddH_2_O.

Thermal cycling included initial denaturation at 98°C for 2 min, followed by 25 cycles consisting of denaturation at 98°C for 15 s, annealing at 55°C for 30 s, and extension at 72°C for 30 s, with a final extension of 5 min at 72°C. PCR amplicons were purified with Agencourt AMPure Beads (Beckman Coulter, Indianapolis, IN, USA) and quantified using the PicoGreen dsDNA Assay Kit (Invitrogen, Carlsbad, CA, USA). After the individual quantification step, amplicons were pooled in equal amounts, and paired-end 2,300-bp sequencing was performed using the Illlumina MiSeq platform with MiSeq Reagent Kit v3 at Shanghai Personal Biotechnology Co., Ltd. (Shanghai, China).

#### Sequence Analysis

The Quantitative Insights Into Microbial Ecology (QIIME, v1.8.0) pipeline was employed to process the sequencing data, as previously described (34). Briefly, raw sequencing reads with exact matches to the barcodes were assigned to respective samples and identified as valid sequences. The low-quality sequences were filtered through following criteria (35, 36) sequences that had a length of <150 bp, sequences that had average Phred scores of <20, sequences that contained ambiguous bases, and sequences that contained mononucleotide repeats of >8 bp. Paired-end reads were assembled using FLASH (37). After chimera detection, the remaining high-quality sequences were clustered into operational taxonomic units (OTUs) at 97% sequence identity by UCLUST (38). A representative sequence was selected from each OTU using default parameters. OTU taxonomic classification was conducted by BLAST searching the representative sequences set against the Greengenes Database (39) using the best hit (40).

### Bioinformatics and Statistical Analysis

Sequence data analyses were mainly performed using QIIME and R packages (v3.2.0). OTU-level alpha diversity indices, such as Chao1 richness estimator, ACE metric (Abundance-based Coverage Estimator), Shannon diversity index, and Simpson index, were calculated using the OTU table in QIIME. OTU-level ranked abundance curves were generated to compare the richness and evenness of OTUs among samples. Differences in the Unifrac distances for pairwise comparisons among groups were determined using Student's *t*-test and the Monte Carlo permutation test with 1,000 permutations. The taxonomy compositions and abundances were visualized using MEGAN (41) and GraPhlAn (42). Venn diagram was generated to visualize the shared and unique OTUs among samples or groups using R package “VennDiagram,” based on the occurrence of OTUs across samples/groups regardless of their relative abundance (43). Taxa abundances at the phylum and genus levels were statistically compared among samples or groups by Metastats (44). Pattern Search was used to identify correlation between the microbial composition of cecal content and dietary supplemental level of vitamin B_12_. Microbial functions were predicted by PICRUSt (phylogenetic investigation of communities by reconstruction of unobserved states), based on high-quality sequences (45). Correlation Heatmap was performed using the OmicStudio tools at https://www.omicstudio.cn/tool.

Data were analyzed with one-way ANOVA by using Statistical Product and Service Solutions (SPSS) 22.0 (SPSS Inc., Chicago, IL, USA). The significant difference of means among treatment groups was identified via Tukey's test. The significance was determined at *P* < 0.05.

## Results

### Production Performance, Egg Quality, and Serum Indicators of Laying Hens

Effects of dietary vitamin B_12_ on the production performance of laying hens are shown in Table 2. There was no significant difference among all treatments for egg production, feed intake, feed conversion ratio, egg mass, and egg weight (*P* > 0.05).

**Table 2:** Effects of dietary supplementation of vitamin B_12_ on the production performance of laying hens^*^.

**Items**		**Vitamin B_12_ level (μg/kg)**				**SEM**	***P*** **-Value**
		**0**	**25**	**100**	**400**		
Egg production (%)	Adjustment period	91.49	92.31	91.22	92.41	0.62	0.73
	Experimental period	90.63	91.87	90.34	91.85	1.25	0.81
Feed intake (g/hen/day)	122.1	124.9	123.3	123.3	2.18	0.91	
Feed conversion ratio (feed/egg mass)	2.10	2.13	2.14	2.10	0.06	0.65	
Egg mass (g/hen/day)	58.20	58.73	57.75	58.79	0.89	0.44	
Egg weight (g)	64.22	63.93	63.92	64.01	0.67	0.60	

* *Data are expressed as the means and pooled standard error of the mean (SEM) (n = 6)*.

Table 3 lists the effect of dietary vitamin B_12_ on egg quality parameters. The shell percentage of eggs from hens fed diet supplemented with 400 μg/kg of vitamin B_12_ was higher (*P* < 0.05) than that of eggs from hens fed other treatment diets. The shell thickness of eggs from hens fed diet supplemented with 100 μg/kg of vitamin B_12_ was higher (*P* < 0.05) than that of eggs from hens fed control diet or supplemented with 25 μg/kg of vitamin B_12_. No significant effect of dietary treatments on other egg quality parameters was detected (*P* > 0.05).

**Table 3:** Effects of dietary supplementation of vitamin B_12_ on the eggshell and egg quality^*^.

**Items**	**Vitamin B_12_ level (μg/kg)**				**SEM**	***P*** **-Value**
	**0**	**25**	**100**	**400**		
Eggshell strength (kgf)	3.752	3.860	4.031	4.231	0.258	0.66
Eggshell/egg weight (%)	10.46^a^	10.57^a^	10.35^a^	11.59^b^	0.002	0.005
Egg yolk/egg weight (%)	25.61	25.03	25.42	25.27	0.124	0.73
Egg white/egg weight (%)	63.93	64.40	64.23	63.14	0.007	0.61
Shape index	1.27	1.27	1.27	1.31	0.014	0.12
Albumen height (mm)	8.58	8.56	8.23	8.32	0.224	0.24
Haugh units	90.89	89.62	88.64	89.43	1.526	0.56
Egg yolk color	5.30	5.50	5.67	5.67	0.419	0.93
Eggshell thickness (mm)	0.362^a^	0.341^a^	0.393^b^	0.373^ab^	0.008	0.002

* *Data are expressed as the means and pooled standard error of the mean (SEM) (n = 6).*

a,b *Means in the same row not sharing a common superscript differ significantly at P < 0.05*.

The biochemical indicators of serum are listed in Table 4. The vitamin B_12_ concentration of serum from hens fed diet with a supplementation of 25 μg/kg of vitamin B_12_ was higher (*P* < 0.05) than that from hens fed diet with no supplementation of vitamin B_12_, but lower (*P* < 0.05) than that from hens fed other two diets with high supplemental levels of vitamin B_12_. There was no significant difference among all treatments for other parameters.

**Table 4:** Effects of dietary supplementation of vitamin B_12_ on the serum indicators of laying hens^*^.

**Items**	**Vitamin B_12_ level (μg/kg)**				**SEM**	***P*** **-Value**
	**0**	**25**	**100**	**400**		
Vitamin B_12_ (ng/ml)	42.28^a^	54.85^b^	76.37^c^	78.93^c^	2.13	0.02
TC (mmol/L)	6.11	6.28	6.00	5.88	0.23	0.57
TG (mmol/L)	11.80	11.98	11.67	12.05	0.82	0.48
Progesterone (ng/ml)	16.53	16.31	17.08	16.58	0.71	0.56
Estrogen (pg/ml)	57.57	58.01	60.07	55.44	1.34	0.60

* *Data are expressed as the means and pooled standard error of the mean (SEM) (n = 6).*

a,b,c *Means in the same row not sharing a common superscript differ significantly at P < 0.05*.

### Cecal Microbiota Composition

**Figure 1:**
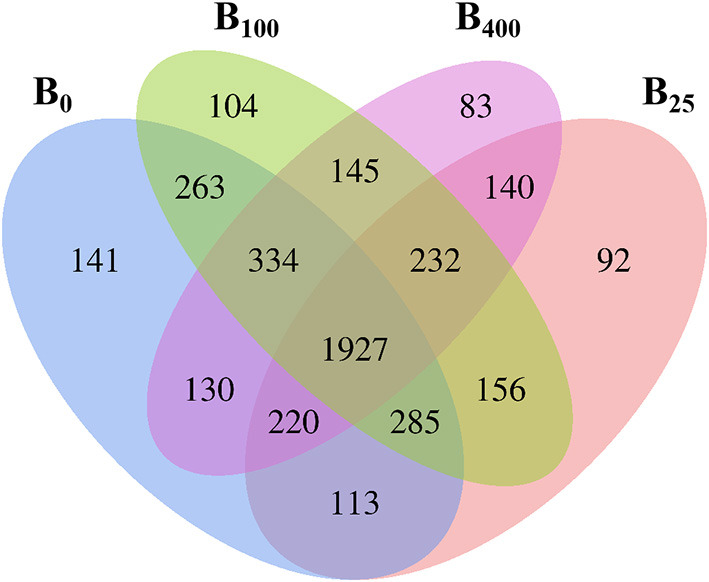
Venn diagram analysis of proportion of the cecal microbes in different treatments.

To explore the diversity of the cecal microbiota of layers after dietary vitamin B_12_ supplementation, the composition and species distribution of the cecal microbiota were investigated by 16S rRNA gene sequencing. After removal of the questioning sequences, a total sequencing quantity was 952,855 that was from 24 samples of cecal content with an average of 39,702 sequences per sample (range from 27,769 to 50,808) for subsequent analysis. A total of 4,386 OTUs after streamlining was characterized into different taxonomic levels including phylum, class, order, family, genus, and species based on Green gene database through QIIME with a 97% species similarity. Venn diagram analysis showed that 1,927 OTUs were shared among the four dietary treatment groups (Figure 1). There were 3,413, 3,165, 3,446, and 3,211 OTUs in treatments B0, B25, B100, and B400, respectively. These results indicated that dietary vitamin B_12_ did not modulate diversity of the cecal microbiota of the layers. However, there were 141, 92, 104, and 83 OUTs that were uniquely identified in four different treatments.

**Figure 2:**
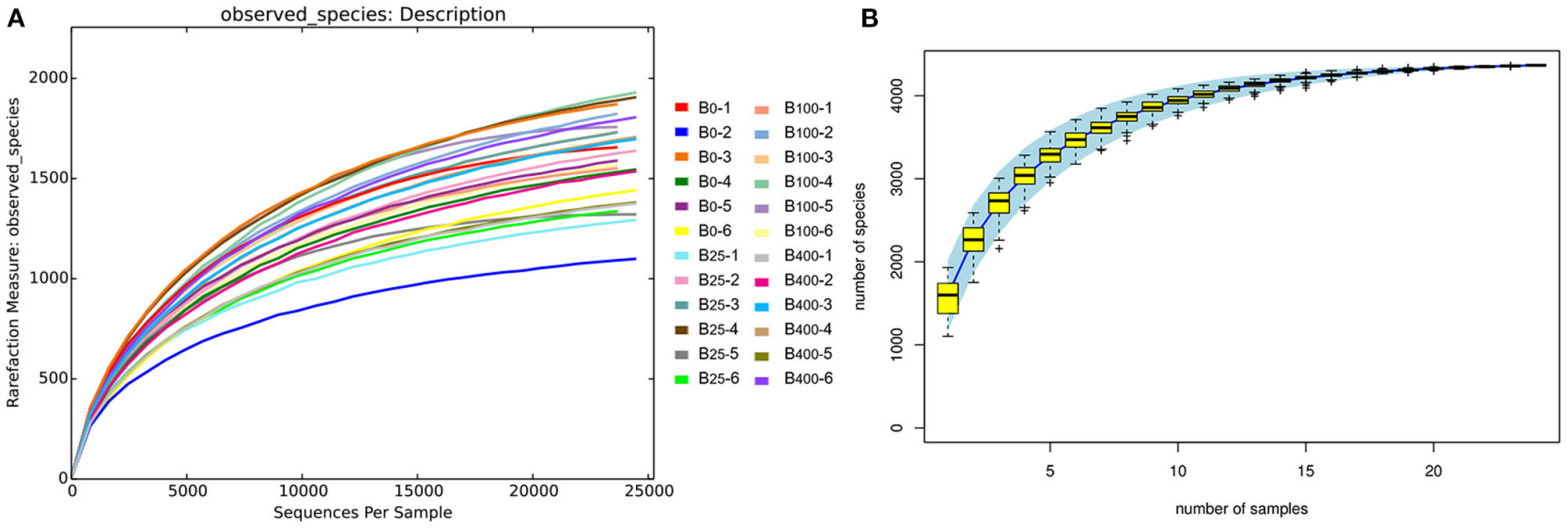
**(A)** Rarefaction curves and **(B)** species accumulation curve diagram analysis of proportion of the cecal microbes in different treatments.

The rarefaction curves and species accumulation curve (Figures 2A,B) for each sample leveled off as the number of sequences increased in all four treatment groups indicating that the samples analyzed had sufficient sequence coverage to accurately describe the bacterial composition of the cecal content in this study.

The alpha diversity of microbiota in the cecal content, including species diversity (Shannon and Simpson indices) and richness (ACE and Chao 1 indices), is shown in Table 5. There was no significant difference among all treatments for these indices, although the group with no vitamin B_12_ supplementation tended to have higher values of ACE and Chao1.

**Table 5:** Effects of dietary supplementation of vitamin B_12_ on the cecal microbial diversity in laying hens^*^.

**Items**	**Vitamin B_12_ level (μg/kg)**				**SEM**	***P*** **-value**
	**0**	**25**	**100**	**400**		
Simpson	0.984	0.987	0.985	0.985	0.002	0.50
Shannon	8.14	8.14	8.03	7.91	0.12	0.51
Chao1	1955.8	1701.2	1696.5	1798.2	104.1	0.36
ACE	2013.8	1768.8	1767.8	1885.8	114.2	0.40

* *Data are expressed as the means and pooled standard error of the mean (SEM) (n = 6). Simpson, Simpson index; Shannon, Shannon diversity index; Chao1, Chao1 richness estimator; ACE, abundance-based coverage estimator metric*.

**Figure 3:**
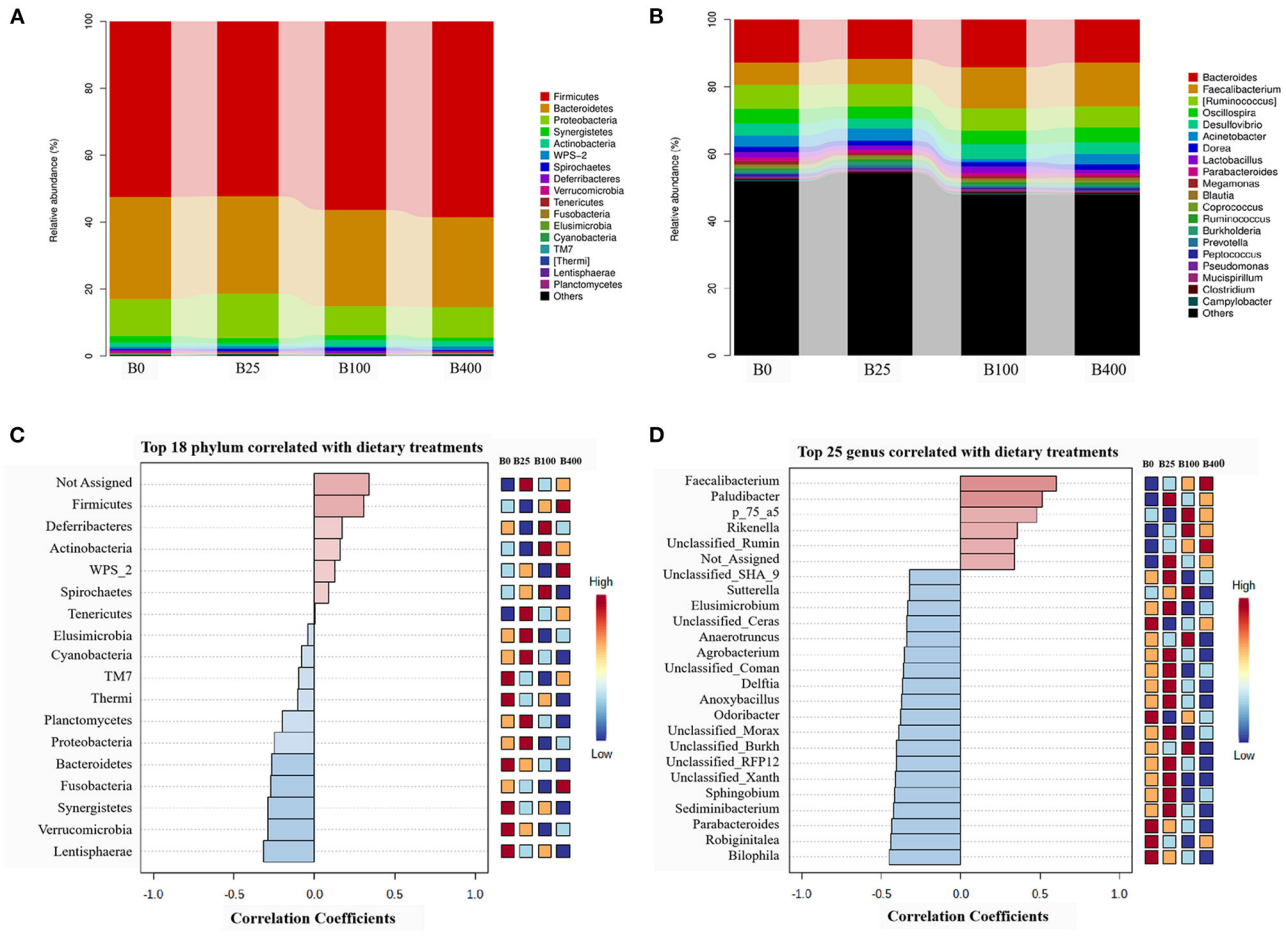
The relative abundance of the cecal microbial composition and correlation with the dietary vitamin B_12_ level at the phylum level **(A,C)** and the genus level **(B,D)**.

Figure 3A and Supplementary Table 1 demonstrated the microbial composition of cecal content from four dietary treatments in the phylum level. The results indicated that cecal microbiota of layers was mainly composed of *Firmicutes, Bacteroidetes*, and *Proteobacteria* accounting for about 94%, with *Firmicutes* being the predominant phylum (>52). A numerical shift in the proportion of *Bacteroidetes* and *Proteobacteria* to *Firmicutes* was observed when high-level vitamin B_12_ (100 or 400 μg/kg) was included in the diets. The proportion of *Firmicutes, Actinobacteria, Deferribacteres, WPS_2*, and *Spirochaetes* is positively correlated, and the proportion of *Elusimicrobia, Thermi, TM7, Cyanobacteria, Lentisphaerae, Planctomycetes, Proteobacteria, Fusobacteria, Verrucomicrobia, Synergistetes*, and *Bacteroidetes* is negatively correlated with the supplemental level of vitamin B_12_ (Figure 3C).

At genus level, a total of 173 species of bacteria in the cecal content of laying hens was observed in this trial, among which 17 genera had the relative abundance of more than 1% (Figure 3B; Supplementary Table 2). Pattern Search results indicated that the abundance of *Butyricicoccus, Faecalibacterium, Paludibacter, p_75_a5*, and *Unclassified_Ruminococcaceae* has a positive correlation, and the abundance of the other 20 bacteria has a negative correlation with the dietary supplemental level of vitamin B_12_ (Figure 3D).

**Figure 4:**
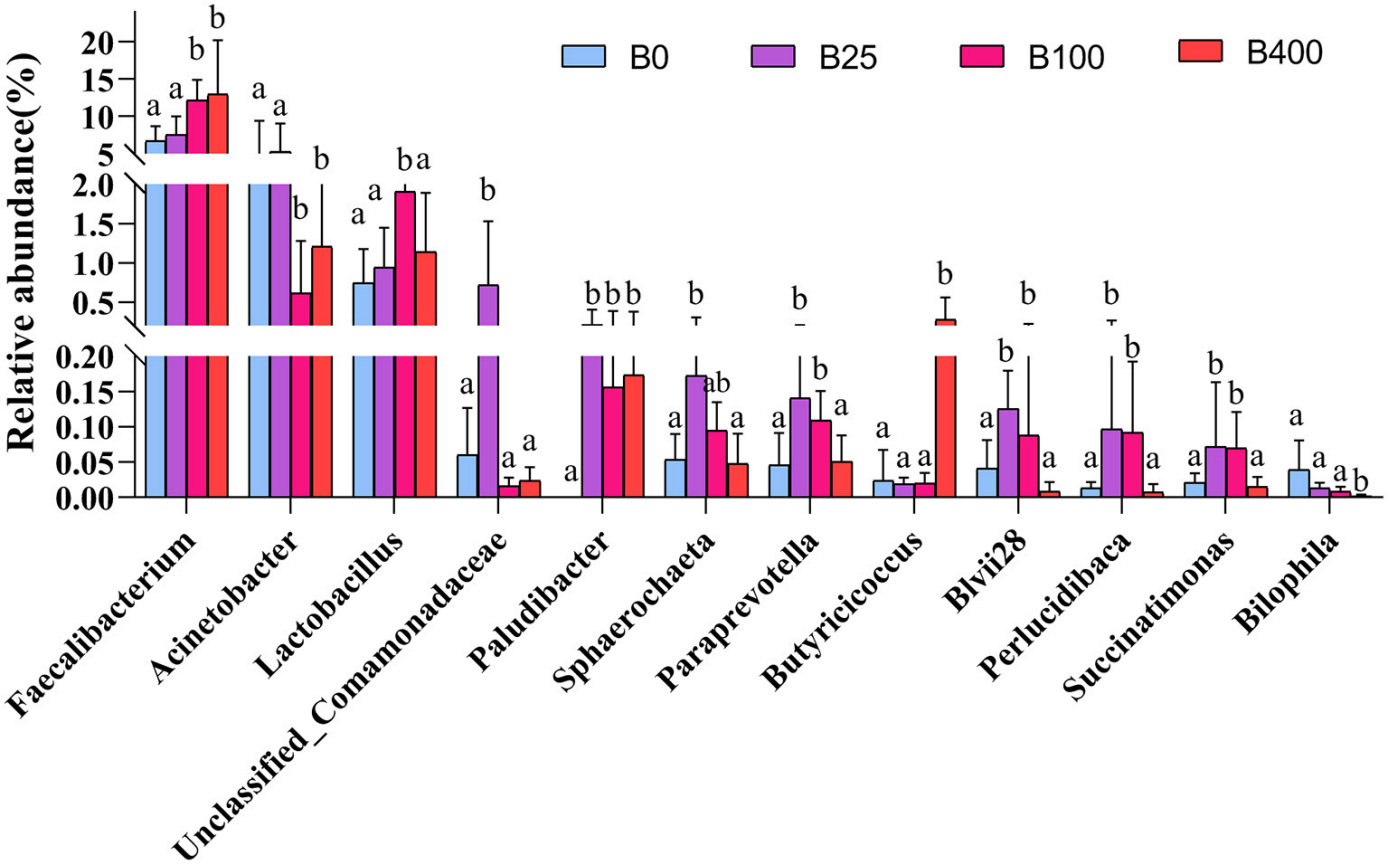
The relative abundance of cecal microbiota of layers fed diets with different levels of vitamin B_12_ at genus level only statistically significant differences are shown. Bars with different superscript letters were significantly different (*P* < 0.05).

As shown in Figure 4, the cecal content from layers fed a diet with supplemental level of 100 or 400 μg/kg of vitamin B_12_ had higher (*P* < 0.01) abundance of *Faecalibacterium* and lower (*P* < 0.05) abundance of *Acinetobacter* compared with the cecal content from layers fed other two diets. The abundance of *Lactobacillu*s in the cecal samples from layers fed 100 μg/kg of a supplemental level of vitamin B_12_ was higher (*P* < 0.05) than that from layers fed with the other three diets. The abundance of *Paraprevotella, Blvii28, Perlucidibaca*, and *Succinatimonas* in the cecal content of the layers fed diets supplemented with 25 or 100 μg/kg of vitamin B_12_ was higher (*P* < 0.05) than that from the layers fed with the other two diets. It was worth noting that *Paludibacter* was not detected in the cecal content of layers fed a diet with no supplementation of vitamin B_12_. The abundance of *Butyricicoccus* was significantly higher, while *Bilophila* was significantly lower in the cecal content of layers fed 400 μg/kg of vitamin B_12_ diet compared with that from layers fed with the other three diets.

### Functional Prediction of the Gut Microbiota

**Figure 5:**
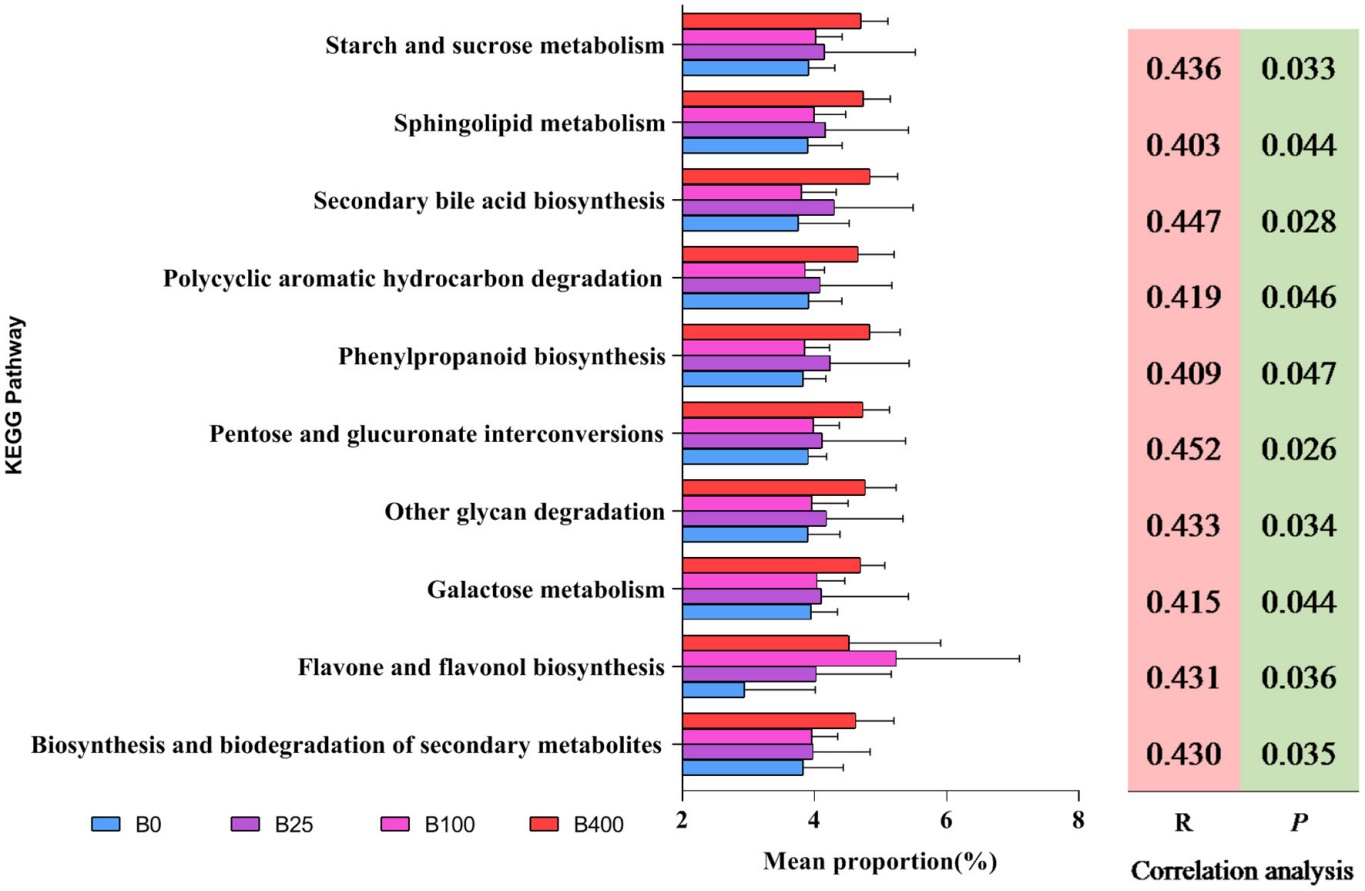
The predicted metabolic attributes of cecal microbiota at genus level from different treatments.

In order to get the predicted metabolic functions of the cecal microbial communities from different treatments, PICRUSt analysis was performed. The results are shown in Figure 5. Ten predicted metabolic functions of the cecal microbial communities were positively correlated with dietary vitamin B_12_ level (*P* < 0.05). These metabolic functions included (1) carbohydrate metabolism such as starch and sucrose metabolism, pentose and glucuronate interconversions, other glycan degradation, and galactose metabolism; (2) lipid metabolism such as sphingolipid metabolism and secondary bile acid biosynthesis; (3) biosynthesis of other secondary metabolites such as phenylpropanoid biosynthesis and flavone and flavonol biosynthesis; (4) other metabolisms such as polycyclic aromatic hydrocarbon degradation and biosynthesis and biodegradation of secondary metabolites. The cecal microbiota from layers fed a diet with 400 μg/kg of vitamin B_12_ increased all the predicted metabolic functions except flavone and flavonol biosynthesis.

**Figure 6:**
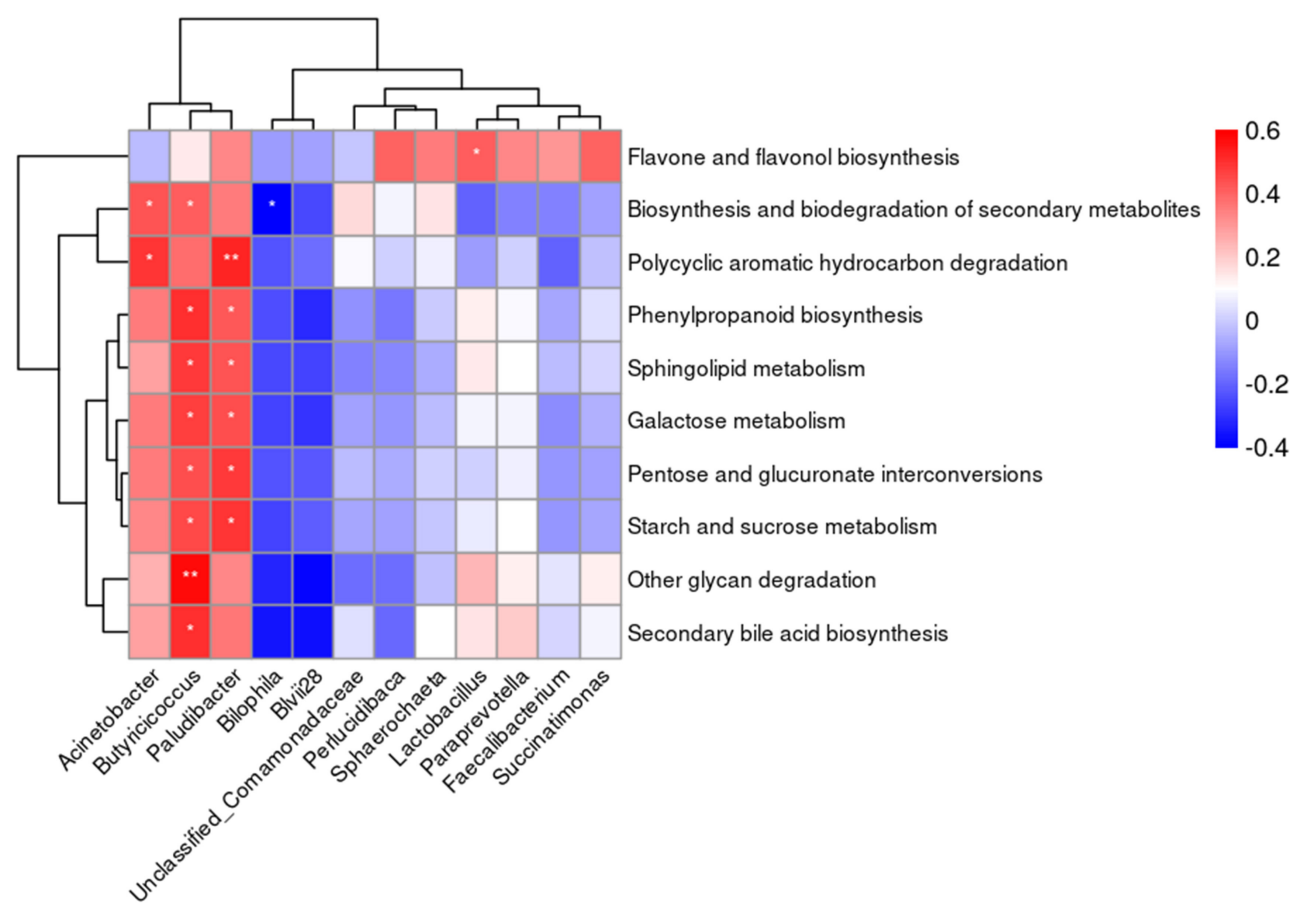
The correlation heatmap between the abundance of microbiota at genus level in the cecal content and the predicted metabolic functions. ^*^P < 0.05, ^**^P < 0.01.

Based on the results of Spearman's analysis (Figure 6), a positive correlation (*P* < 0.05) between the abundance of *Acinetobacter* in the cecal content and the biosynthesis and biodegradation of secondary metabolites and polycyclic aromatic hydrocarbon degradation was found. The abundance of *Butyricicoccus* and *Paludibacter* in the cecal content was positively correlated (*P* < 0.05) with carbohydrate and lipid metabolism. A negative correlation (*P* < 0.05) between the abundance of *Bilophila* in the cecal content and biosynthesis and biodegradation of secondary metabolites was observed. The abundance of *Lactobacillus* was positively correlated (*P* < 0.05) with flavone and flavonol biosynthesis.

## Discussion

The results from the current study showed no significant effect of dietary vitamin B_12_ levels on the production performance of laying hens including egg production, feed intake, and egg weight. Although the concentration of vitamin B_12_ in B0 is below the detectable limit, no negative impact on the production performance due to deficiency of vitamin B_12_ was noticed. This is probably because the layers might get enough reservation of vitamin B_12_ in the tissues from pretrial diets to support the need of production. The similar results were reported by other researchers (46–48). Kominato reported that 2–5 months may be needed to completely deplete hens of vitamin B_12_ stored in tissues (49). The high dietary levels of vitamin B_12_ (100 or 400 μg/kg) used in this trial did not affect the production performance of layers, but significantly increased the thickness and the percentage of eggshell. Leeson and Caston reported that a high dietary vitamin B_12_ had no effect on the egg production of laying hens (15). The improvement of eggshell quality is probably due to the change in microbiota and their metabolites in the gut. A positive correlation between the abundance of butyrate-producing bacterium and *Lactobacillus* in the cecal content and dietary vitamin B_12_ level was found in this trail. Many studies indicated the beneficial effect of dietary inclusion of butyrate or probiotic including *Lactobacillus* on the eggshell quality (50–53).

Vitamin B_12_ is a critical nutrient for human beings, animals, as well as microbes. Studies indicated that dietary inclusion of vitamin B_12_ increased the level of vitamin B_12_ in the hind gut and modulated the structure and function of microbial communities in humans and mice (22, 54, 55). In the current study, although different dietary levels of vitamin B_12_ did not alter the diversity of cecal microbiota of layer hens as evident by the similar α-diversity index among different treatments, the community structure and abundance of the microbiota in the cecum were significantly changed. A positive correlation between dietary level of vitamin B_12_ and cecal abundance of *Firmicutes* was detected. Studies found that *Firmicutes* is one of the dominant microorganisms in the cecum of chicken, and the abundance of *Firmicutes* is beneficial to the health of the bird because of its anti-inflammatory effects (56–58). The negative correlation between the dietary level of vitamin B_12_ and the richness of *Bacteroidetes* in the cecal content observed in this trial did not support the results reported by Kelly et al. and Wang (55, 59). The abundance changes in microbiota due to dietary vitamin B_12_ level could impact the energy metabolism of laying hens. This is based on some evidence showing the close relationship between the abundance of *Firmicutes* or *Bacteroidetes* and body fat accumulation in humans and some obesity-related parameters in mice (60, 61). However, further investigation is needed.

It is well-documented that butyrate can positively impact intestinal health through the synergistic effect of providing energy to the intestinal cell and anti-inflammatory effect (62–64). Major butyrate-producing bacteria in the gastral intestinal tract included *F. prasusnitzii* in *Faecalibacterium* genus and *Butyricoccus* (65, 66). They all played a very important role in host intestinal health (58, 66–68). Results from the current study indicated that *Faecalibacterium* was the core genus in the cecum of laying hens and was positively correlated with dietary supplemental levels of vitamin B_12_, and dietary supplementation of high-level vitamin B_12_ (100 or 400 μg/kg) increased the abundance of *Butyricoccus*. The increased abundance of *Lactobacillus, Paraprevotella, Succinatimonas, Paludibacter*, and *Sphaerochaeta* in the cecal content due to high dietary vitamin B_12_ (100 or 400 μg/kg) observed in this trial could also positively impact the gut health of laying hens. This is because bacteria in the gut, including *Lactobacillus* and *Paraprevotella*, have been proven to play an active and protective role in intestinal health of the host through producing antimicrobial molecules, competitive exclusion, and changing bile acid metabolism in the gut (69–71). *Succinatimonas, Paludibacter*, and *Sphaerochaeta* were found to produce volatile fatty acids including acetic acid, propionic acid, and succinic acid that could positively impact energy metabolism of the host and regulate hindgut pH to inhibit the growth of harmful bacteria (72–77). Dietary supplementation of high-level vitamin B_12_ depressed the richness of *Acinetobacter* and *Bilophila* that were inflammation-associated genera (78). The composition changes in cecal microbiota of laying hens found in this trial due to high dietary vitamin B_12_ was also significantly correlated to the predicted metabolism function of microbial communities in the cecum including carbohydrate, lipid, and many other metabolisms. Similar results were reported by other researchers (79).

## Conclusions

To our knowledge, this is the first trial to explore the relationship between high dietary levels of vitamin B_12_ and the cecal microbiota in laying hens. The results indicated that dietary supplementation of 100 μg/kg of vitamin B_12_ had equivalent effects with the supplemental level of 400 μg/kg. Both levels caused a significant change in composition and metabolic functions of cecal microorganisms, which could positively impact eggshell quality, metabolism, and gut health of laying hens during peak production period. These findings provided the valuable information for the application of high supplemental levels of vitamin B_12_ in the diet of laying hens for production or health purposes.

## Data Availability Statement

The datasets presented in this study can be found in online repositories. The names of the repository/repositories and accession number(s) can be found below: BioProject ID PRJNA732929.

## Ethics Statement

All experimental procedures were carried out in accordance with the Guidelines of the Shanxi Agricultural University Animal Experiment Ethics Committee, and the license number was SXAU-EAW-2017-002Chi.001.

## Author Contributions

YY: conceptualized the study, supervised the study, was in charge of the project administration, and acquired the funding. RW: developed the methodology, performed the formal analysis and data curation, obtained the resources, and wrote and prepared the original draft. RRL and RW: conducted the investigation. RW, XTW, and YB wrote, reviewed, and edited the manuscript. All authors have read and agreed to the published version of the manuscript.

## Funding

This research was funded by the 1331 Key Discipline Construction of Engineering Animal Husbandry of Shanxi Province (J202011315), Key Research and Development Project of Science and Technology (Agriculture) of Jinzhong City (Y182011), and Scientific and Technological Innovation Programs of Higher Education Institutions in Shanxi (2019L0959).

## Conflict of Interest

The authors declare that the research was conducted in the absence of any commercial or financial relationships that could be construed as a potential conflict of interest.

## Publisher's Note

All claims expressed in this article are solely those of the authors and do not necessarily represent those of their affiliated organizations, or those of the publisher, the editors and the reviewers. Any product that may be evaluated in this article, or claim that may be made by its manufacturer, is not guaranteed or endorsed by the publisher.
