# Supplementary material for: A Comparison of Production Performance, Egg Quality, and Cecal Microbiota in Laying Hens Receiving Graded Levels of Vitamin B12
Source: Front Vet Sci. 2021 Oct 21;8:712183. doi: 10.3389/fvets.2021.712183 (PMC8566728; doi:10.3389/fvets.2021.712183)
Supplement: Supplementary file 1 [file Data_Sheet_1.docx]

Supplementary Material

# Supplementary Tables

Supplementary Table S1 Relative abundance of phylum level communities

| **Taxon** | **0** | **25** | **100** | **400** |
| --- | --- | --- | --- | --- |
| p__Firmicutes | 52.5% | 52.3% | 56.4% | 58.5% |
| p__Bacteroidetes | 30.5% | 29.1% | 28.8% | 26.9% |
| p__Proteobacteria | 11.1% | 13.3% | 8.6% | 9.1% |
| p__Synergistetes | 2.0% | 1.5% | 1.6% | 1.1% |
| p__Actinobacteria | 1.2% | 0.9% | 1.8% | 1.6% |
| p__WPS-2 | 0.7% | 0.8% | 0.4% | 1.1% |
| p__Spirochaetes | 0.3% | 0.7% | 1.0% | 0.3% |
| No blast hit;Other | 0.3% | 0.5% | 0.4% | 0.4% |
| p__Deferribacteres | 0.3% | 0.2% | 0.7% | 0.3% |
| p__Verrucomicrobia | 0.5% | 0.1% | 0.1% | 0.1% |
| p__Tenericutes | 0.1% | 0.2% | 0.2% | 0.2% |
| p__Fusobacteria | 0.2% | 0.1% | 0.0% | 0.2% |
| p__Elusimicrobia | 0.1% | 0.2% | 0.1% | 0.1% |
| p__Cyanobacteria | 0.1% | 0.1% | 0.1% | 0.1% |
| p__TM7 | 0.1% | 0.1% | 0.0% | 0.1% |
| p__[Thermi] | 0.0% | 0.0% | 0.0% | 0.0% |
| p__Lentisphaerae | 0.0% | 0.0% | 0.0% | 0.0% |
| p__Planctomycetes | 0.0% | 0.0% | 0.0% | 0.0% |

Supplementary Table S2 Relative abundance of genus level communities

| **Taxon** | **0** | **25** | **100** | **400** |
| --- | --- | --- | --- | --- |
| g__Bacteroides | 12.86% | 11.75% | 14.26% | 12.85% |
| g__Unclassified_Ruminococcaceae | 9.74% | 10.95% | 10.96% | 11.27% |
| g__Faecalibacterium | 6.68% | 7.57% | 12.19% | 12.97% |
| g__Unclassified_Bacteroidales | 8.56% | 8.86% | 6.10% | 8.07% |
| g__Unclassified_Clostridiales | 6.17% | 7.88% | 6.04% | 6.74% |
| g__[Ruminococcus] | 7.01% | 6.56% | 6.64% | 6.26% |
| g__Unclassified_Veillonellaceae | 6.05% | 4.20% | 5.18% | 4.89% |
| g__Oscillospira | 4.29% | 3.57% | 4.02% | 4.50% |
| g__Desulfovibrio | 3.67% | 2.98% | 4.34% | 3.38% |
| g__Unclassified_S24-7 | 3.33% | 3.24% | 4.12% | 1.96% |
| g__Acinetobacter | 3.37% | 3.57% | 0.94% | 3.08% |
| g__Unclassified_Lachnospiraceae | 2.30% | 2.86% | 2.55% | 2.56% |
| g__Unclassified_Synergistaceae | 1.97% | 1.52% | 1.55% | 1.06% |
| g__Dorea | 1.60% | 1.45% | 1.36% | 1.66% |
| g__Lactobacillus | 1.57% | 1.28% | 1.91% | 1.14% |
| g__Unclassified_Coriobacteriaceae | 0.95% | 0.70% | 1.46% | 1.30% |
| g__Parabacteroides | 1.01% | 1.00% | 0.92% | 0.73% |
| g__Unclassified_WPS-2 | 0.66% | 0.75% | 0.39% | 1.06% |
| g__Megamonas | 0.98% | 0.55% | 0.74% | 0.43% |
| f__Lachnospiraceae;g__Blautia | 0.64% | 0.56% | 0.58% | 0.89% |
| g__Coprococcus | 0.60% | 0.82% | 0.54% | 0.66% |
| g__Unclassified_BS11 | 1.56% | 0.23% | 0.50% | 0.32% |
| g__Ruminococcus | 0.56% | 0.72% | 0.69% | 0.62% |
| g__Burkholderia | 0.68% | 0.93% | 0.29% | 0.50% |
| g__Unclassified_Christensenellaceae | 1.15% | 0.47% | 0.24% | 0.48% |
| g__Prevotella | 0.46% | 0.77% | 0.59% | 0.49% |
| g__Peptococcus | 0.68% | 0.29% | 0.41% | 0.67% |
| g__Unclassified_Rikenellaceae | 0.71% | 0.45% | 0.37% | 0.45% |
| g__Unclassified_[Paraprevotellaceae] | 0.40% | 0.77% | 0.40% | 0.34% |
| g__Unclassified_Bacteroidaceae | 0.38% | 0.38% | 0.29% | 0.51% |
| Other | 0.27% | 0.46% | 0.39% | 0.43% |
| g__Pseudomonas | 0.40% | 0.61% | 0.17% | 0.35% |
| g__Mucispirillum | 0.31% | 0.22% | 0.69% | 0.29% |
| g__Clostridium | 0.32% | 0.34% | 0.34% | 0.44% |
| g__Unclassified_Clostridiaceae | 0.33% | 0.39% | 0.35% | 0.30% |
| g__Unclassified_Burkholderiales | 0.35% | 0.56% | 0.13% | 0.21% |
| g__Campylobacter | 0.39% | 0.18% | 0.48% | 0.17% |
| g__Megasphaera | 0.24% | 0.36% | 0.36% | 0.27% |
| g__Unclassified_[Barnesiellaceae] | 0.28% | 0.41% | 0.25% | 0.24% |
| g__Treponema | 0.15% | 0.43% | 0.43% | 0.15% |
| g__Sutterella | 0.29% | 0.31% | 0.32% | 0.17% |
| g__Stenotrophomonas | 0.31% | 0.32% | 0.14% | 0.24% |
| g__Odoribacter | 0.33% | 0.19% | 0.20% | 0.19% |
| g__Coprobacillus | 0.17% | 0.19% | 0.29% | 0.25% |
| g__Unclassified_Erysipelotrichaceae | 0.20% | 0.14% | 0.20% | 0.33% |
| g__Unclassified_Peptostreptococcaceae | 0.31% | 0.24% | 0.13% | 0.14% |
| g__Unclassified_Comamonadaceae | 0.06% | 0.72% | 0.02% | 0.02% |
| g__Cupriavidus | 0.24% | 0.21% | 0.16% | 0.14% |
| g__Unclassified_Betaproteobacteria | 0.06% | 0.36% | 0.25% | 0.03% |
| g__Unclassified_Spirochaetaceae | 0.08% | 0.06% | 0.45% | 0.08% |
| g__Unclassified_RF39 | 0.13% | 0.23% | 0.15% | 0.16% |
| g__Unclassified_Rhodocyclaceae | 0.10% | 0.20% | 0.32% | 0.02% |
| g__Akkermansia | 0.42% | 0.06% | 0.05% | 0.11% |
| g__Unclassified_[Mogibacteriaceae] | 0.16% | 0.15% | 0.16% | 0.16% |
| g__cc_115 | 0.18% | 0.15% | 0.14% | 0.14% |
| g__Collinsella | 0.12% | 0.07% | 0.23% | 0.15% |
| g__Fusobacterium | 0.21% | 0.06% | 0.04% | 0.24% |
| g__Ochrobactrum | 0.13% | 0.26% | 0.08% | 0.07% |
| g__Ralstonia | 0.10% | 0.26% | 0.11% | 0.07% |
| g__Paludibacter | 0.00% | 0.21% | 0.16% | 0.17% |
| g__Unclassified_Prevotellaceae | 0.10% | 0.15% | 0.05% | 0.15% |
| g__Unclassified_Elusimicrobiaceae | 0.10% | 0.14% | 0.07% | 0.09% |
| g__Unclassified_Desulfovibrionaceae | 0.11% | 0.11% | 0.13% | 0.05% |
| g__Unclassified_YS2 | 0.09% | 0.13% | 0.08% | 0.08% |
| g__Brevundimonas | 0.09% | 0.16% | 0.05% | 0.08% |
| g__[Eubacterium] | 0.09% | 0.06% | 0.08% | 0.14% |
| g__Sphaerochaeta | 0.05% | 0.17% | 0.09% | 0.05% |
| g__Unclassified_RF32 | 0.06% | 0.14% | 0.11% | 0.05% |
| g__Herbaspirillum | 0.11% | 0.13% | 0.04% | 0.08% |
| g__Paraprevotella | 0.05% | 0.14% | 0.11% | 0.05% |
| g__Barnesiella | 0.05% | 0.13% | 0.11% | 0.06% |
| g__Butyricicoccus | 0.02% | 0.02% | 0.02% | 0.28% |
| g__Enterococcus | 0.06% | 0.13% | 0.01% | 0.13% |
| g__Unclassified_Caulobacteraceae | 0.05% | 0.20% | 0.04% | 0.03% |
| g__Helicobacter | 0.02% | 0.16% | 0.10% | 0.01% |
| g__Blvii28 | 0.04% | 0.13% | 0.09% | 0.01% |
| g__Slackia | 0.09% | 0.03% | 0.06% | 0.06% |
| g__Unclassified_Alphaproteobacteria | 0.03% | 0.13% | 0.03% | 0.03% |
| g__Unclassified_Rs-045 | 0.08% | 0.05% | 0.02% | 0.07% |
| g__Perlucidibaca | 0.01% | 0.10% | 0.09% | 0.01% |
| g__Unclassified_RFP12 | 0.09% | 0.07% | 0.02% | 0.03% |
| g__Butyricimonas | 0.04% | 0.02% | 0.02% | 0.12% |
| g__Elizabethkingia | 0.08% | 0.04% | 0.06% | 0.03% |
| g__Unclassified_Moraxellaceae | 0.03% | 0.12% | 0.02% | 0.03% |
| g__Dehalobacterium | 0.06% | 0.03% | 0.03% | 0.06% |
| g__Agrobacterium | 0.06% | 0.08% | 0.03% | 0.02% |
| g__Sphingobacterium | 0.06% | 0.07% | 0.02% | 0.03% |
| g__Succinatimonas | 0.02% | 0.07% | 0.07% | 0.01% |
| g__Turicibacter | 0.06% | 0.06% | 0.02% | 0.02% |
| g__Alistipes | 0.07% | 0.04% | 0.03% | 0.03% |
| g__Aeriscardovia | 0.05% | 0.01% | 0.03% | 0.05% |
| g__YRC22 | 0.06% | 0.03% | 0.02% | 0.04% |
| g__Unclassified_[Chromatiaceae] | 0.03% | 0.05% | 0.02% | 0.03% |
| g__Unclassified_F16 | 0.05% | 0.03% | 0.02% | 0.03% |
| g__Unclassified_Bradyrhizobiaceae | 0.02% | 0.05% | 0.02% | 0.02% |
| g__Tannerella | 0.03% | 0.03% | 0.03% | 0.02% |
| g__Anaerovorax | 0.06% | 0.02% | 0.01% | 0.01% |
| g__Unclassified_Enterobacteriaceae | 0.02% | 0.04% | 0.00% | 0.03% |
| g__Unclassified_SHA-98 | 0.03% | 0.03% | 0.01% | 0.02% |
| g__Streptococcus | 0.01% | 0.07% | 0.00% | 0.01% |
| g__Roseburia | 0.02% | 0.03% | 0.02% | 0.01% |
| g__Sediminibacterium | 0.02% | 0.04% | 0.01% | 0.01% |
| g__Brevibacterium | 0.00% | 0.08% | 0.00% | 0.00% |
| g__Unclassified_Rhizobiales | 0.01% | 0.03% | 0.01% | 0.01% |
| g__Xanthobacter | 0.03% | 0.01% | 0.02% | 0.02% |
| g__Anaerotruncus | 0.02% | 0.01% | 0.03% | 0.01% |
| g__[Prevotella] | 0.00% | 0.00% | 0.06% | 0.00% |
